# Key factors for differential drought tolerance in two contrasting wild materials of *Artemisia wellbyi* identified using comparative transcriptomics

**DOI:** 10.1186/s12870-022-03830-3

**Published:** 2022-09-17

**Authors:** Huan Liu, Qiyu Wang, Jinglong Wang, Yunfei Liu, Wangdui Renzeng, Guiqin Zhao, Kuiju Niu

**Affiliations:** 1 Key Laboratory of Grassland Ecosystems, College of Grassland Science, Ministry of Education, Gansu Agricultural University, Lanzhou, 730070 China; 2 Tibet Grassland Science Research Institute, Tibet Academy of Agricultural and Animal Husbandry Sciences, Lhasa, 850000 China

**Keywords:** Tibetan Plateau, *Artemisia wellbyi*, Drought stress, Transcriptional regulation

## Abstract

**Background:**

Drought is a significant condition that restricts vegetation growth on the Tibetan Plateau. *Artemisia wellbyi* is a unique semi-shrub-like herb in the family Compositae, which distributed in northern and northwest of Tibetan Plateau. It is a dominant species in the community that can well adapt to virous environment stress, such as drought and low temperature. Therefore, *A. wellbyi*. has a potential ecological value for soil and water conservation of drought areas. Understanding the molecular mechanisms of *A. wellbyi*. that defense drought stress can acquire the key genes for drought resistance breeding of *A. wellbyi*. and provide a theoretical basis for vegetation restoration of desertification area. However, they remain unclear. Thus, our study compared the transcriptomic characteristics of drought-tolerant “11” and drought-sensitive “6” material of *A. wellbyi* under drought stress.

**Results:**

A total of 4875 upregulated and 4381 downregulated differentially expressed genes (DEGs) were induced by drought in the tolerant material; however, only 1931 upregulated and 4174 downregulated DEGs were induced by drought in the sensitive material. The photosynthesis and transcriptional regulation differed significantly with respect to the DEGs number and expression level. We found that CDPKs (calmodulin-like domain protein kinases), SOS3 (salt overly sensitive3), MAPKs (mitogen-activated protein kinase cascades), RLKs (receptor like kinase), and LRR-RLKs (repeat leucine-rich receptor kinase) were firstly involved in response to drought stress in drought tolerant *A. wellbyi*. Positive regulation of genes associated with the metabolism of ABA (abscisic acid), ET (ethylene), and IAA (indole acetic acid) could play a crucial role in the interaction with other transcriptional regulatory factors, such as MYBs (v-myb avian myeloblastosis viral oncogene homolog), AP2/EREBPs (APETALA2/ethylene-responsive element binding protein family), WRKYs, and bHLHs (basic helix-loop-helix family members) and receptor kinases, and regulate downstream genes for defense against drought stress. In addition, HSP70 (heat shock protein70) and MYB73 were considered as the hub genes because of their strong association with other DEGs.

**Conclusions:**

Positive transcriptional regulation and negative regulation of photosynthesis could be associated with better growth performance under drought stress in the drought-tolerant material. In addition, the degradation of sucrose and starch in the tolerant *A. wellbyi* to alleviate osmotic stress and balance excess ROS. These results highlight the candidate genes that are involved in enhancing the performance of drought-tolerant *A. wellbyi* and provide a theoretical basis for improving the performance of drought-resistant *A. wellbyi*.

**Supplementary Information:**

The online version contains supplementary material available at 10.1186/s12870-022-03830-3.

## Introduction

The Tibetan Plateau is the most important climate regulator in the world, with abundant aeolian sandy lands, sparse vegetation, low temperature, and an average elevation of over 4000 m [1, 2]. The ecosystem in this region is sensitive to external disturbance because of the hindrance in water source accumulation induced by windy and aeolian sandy lands conditions [3]. Desertification induced by drought conditions is recognized as one of the main environmental issues in terms of soil and water conservation and biodiversity protection [4]. Drought can decrease vegetation productivity, accelerate alpine grassland degradation, and pose challenges to sustainable development [5]. Therefore, germplasm resources should be explored and improved to increase vegetation restoration and reduce plant mortality in extreme drought conditions.

Protein kinases and hormones are the early responders to drought stress in plants [6, 7]. ABA is the main regulator involved in regulating the stomatal closure under drought stress [8]. ABA-responsive elements (ABREs) are required for drought stress response to binding with *cis*-acting elements for initiating the transcription of downstream related genes [9]. The ABRE-binding protein EmBP-1 can encode a basic leucine zipper (bZIP) protein that involved in ABA-dependent signal transduction pathway under drought stress [10]. AREB transcription factors are activated by ABA through the multisite phosphorylation [11]. For ABA-independent pathway, AP2/ERF family members *DREB2A* and *DREB2B* were reported that involved in response drought stress [12]. The GROWTH-REGULATING FACTOR7 (GRF7) as a negative regulator inhibits the expression of *DREB2A* under normal growth conditions, and *GRF7* knockout and knockdown plants increased the expression levels of osmotic stress-responsive genes [11]. A ubiquitin E3 ligase DREB2A-INTERACTING PROTEIN1 (DRIP1) involves in degradation of DREB2A protein under unstress condition in plants, whereas the *drip1* and *drip2* mutant appeared a higher resistance of drought [13]. The Ca^2+^-dependent signal transduction pathway also plays a pivotal role in responding drought stress. Ca^2+^-dependent protein kinases (CDPKs) are the major sensors that translate Ca^2+^ signals into phosphorylation events [14]. CDPK2 can increase the jasmonic acid (JA) and ethylene (ET) concentration and trigger enhanced levels of JA and ET response genes [15]. Transcription factors (TFs) induced by signal transduction pathways regulate downstream stress-related genes, leading to variation in protein abundance and metabolism to protect cell membranes [16]. Chen [17] showed that the transcription factor *GbMYB5* positively regulates drought stress in *Gossypium barbadense.* The transcription factor *AtWRKY30* can enhance tolerance to drought in *Triticum aestivum* by increasing the rate of gas exchange and the relative water content in leaves [18]. bZIP [19], NAC [20, 21], dehydration-responsive binding protein (DREB) [22], ERF [23], bHLH [24], are also involved in increasing drought stress tolerance. At the physiological level, water use efficiency-related characteristics, such as the closure of stomata and uptake of nutrients, would be regulated to alleviate the damage caused by drought [25]. Moreover, key enzymes related to low-molecular-weight osmolytes, such as glycine betaine, proline, and organic acids, were significantly up/downregulated to enhance water retention and absorption [26].

*Artemisia wellbyi* is one of the most important drought-tolerant species in the Tibetan Plateau because of its wide distribution and adaptation to various stresses [27]. It is the primary plant foraged by livestock in autumn and winter. However, its growth is limited by the drought conditions of the Tibetan Plateau [28]. Although many molecular experiments have been conducted to reveal the extreme drought response mechanisms of plants, few mechanisms have been identified in *A. wellbyi*, particularly as they are related to the molecular response of *A. wellbyi* to extreme drought.

RNA-seq technology is routinely used to identify DEGs genes (differentially expressed) involved in defense reactions and plant development. In recent years, genes associated with drought resistance have been identified, including ABA signaling genes [29], water transport-related protein aquaporin (*AQP*) [30], antioxidant defense genes such as ascorbate peroxidase (*APX*), superoxide dismutase (*SOD*), peroxidase (*POD*), and catalase (*CAT*) [31], and macro-molecule transporters such as heat shock proteins (*Hsp70*, *Hsf8-like*, *HSP70/DNAK*) [32]. In this study, weperformed comparative transcriptomics to explain the different defense reactions between drought-tolerant and drought-sensitive *A. wellbyi* materials under drought stress. There were two main objectives: 1) to investigate the essential pathways involved in response to drought stress and 2) to ascertain the key stress-responsive TFs and predict the protein–protein interaction (PPI) network and hub genes that respond to drought stress.

## Results

### Global variations of phenotype and DEGs under drought stress

**Fig. 1:**
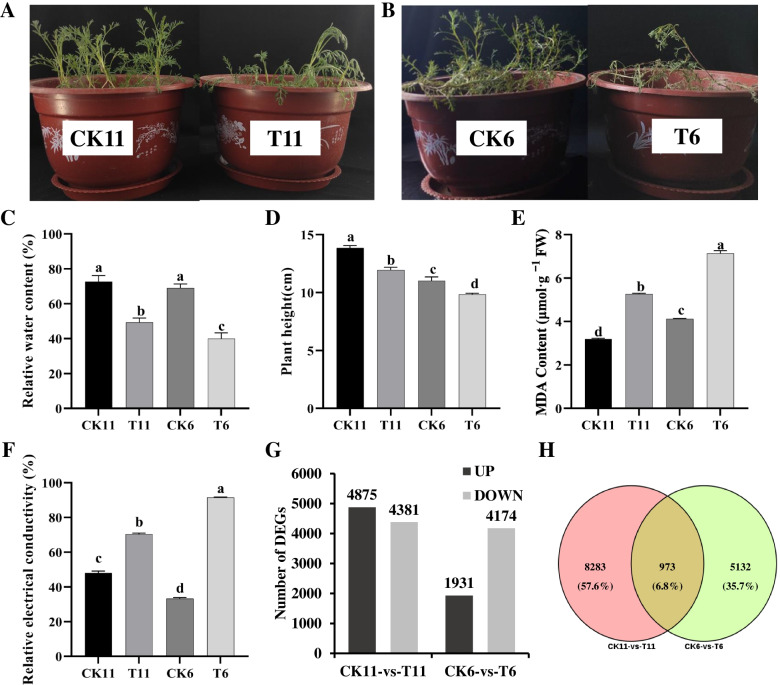
Comparisons of phenotype (**A**, **B**), relative water content (RWC, **C**), plant height (**D**), malondialdehyde (MDA, **E**) content, and relative electrical conductivity (REC, **F**), DEGs (**G**) and Venn diagram (**H**) between drought-tolerant and drought-sensitive materials under drought stress. CK11, well-watered drought-tolerant material; T11, drought-tolerant material exposed to drought stress; CK6, well-watered drought-sensitive material; T6, drought-sensitive material exposed to drought stress; different letters represent a significant difference (*p* < 0.05) between four treatments

A better performance of phenotype was observed in drought tolerant *A. wellbyi* after a 30 d of drought stress (Fig. 1A–B). The RWC and plant height were significantly inhibited in both materials, but the RWC and plant height of the tolerant material”11” was significantly higher than the sensitive material “6” after drought stress (Fig. 1C–D). Furthermore, the MDA content and REC were significantly increased with drought. The MDA content and REC in the sensitive material “6″ were remarkably higher than in the tolerant material “11″ under drought stress (Fig. 1E–F).

A total of 701,181,204 clean reads and 104,423,163,740 bp were generated from 12 samples after filtering (Table S2, 3). A total of 175,684 genes with an average length of 769 bp and 39.81% GC content were generated after *de novo* assembly (Table S4). A total of 9256 (4875 upregulated and 4381 down-regulated) and 6106 (1931 up-regulated and 4174 down-regulated) DEGs were involved in the response to drought stress in both materials, respectively (Fig. 1G). A total of 973 (6.8%) DEGs were significantly regulated by drought stress in both materials (Fig. 1H). A total of 20 DEGs were selected to identify the accuracy of RNA-seq, and the Pearson’s correlation coefficient (R^2^) of Log2FC in RNA-Seq and RT-qPCR is 0.9461 (Table S1). This result showed that the expression levels of DEGs is consistent regardless of the investigation method.

### GO classification of DEGs

**Fig. 2:**
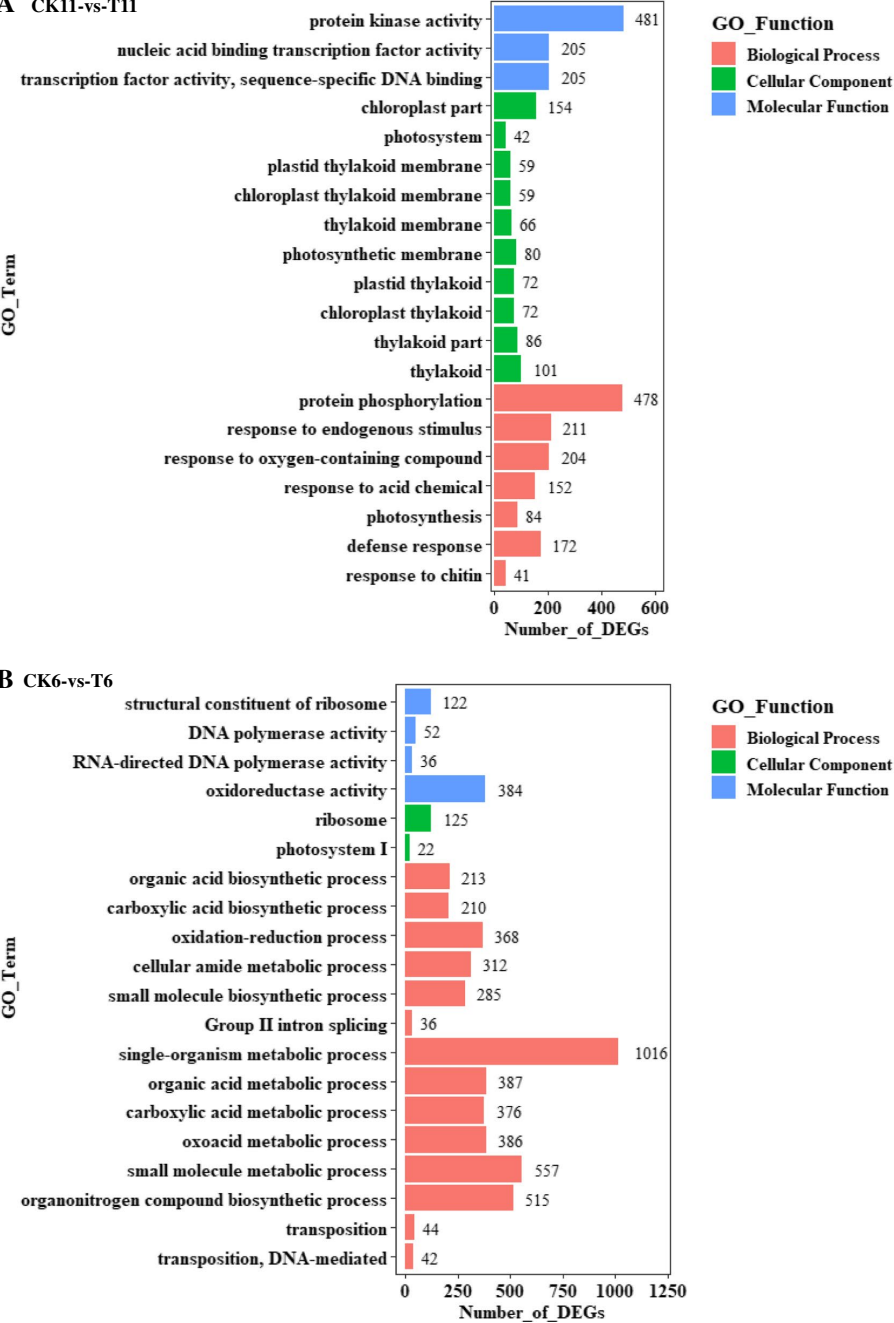
Significantly enriched GO terms in leaves of *Artemisia wellbyi* when exposed to drought compared with CK. **A** Significantly enriched GO terms in drought-tolerant material. **B** Significantly enriched GO terms in drought-sensitive material. CK11, well-watered drought-tolerant material; T11, drought-tolerant material exposed to drought stress; CK6, well-watered drought-sensitive material; T6, drought-sensitive material exposed to drought stress

The DEGs of 11 and 6 were mapped to the GO database to classify the function of DEGs and identify the significantly enriched GO terms. The top 20 significantly enriched GO terms ranked by *p*-value were considered to be highly related to drought stress response. The results showed that 7 and 14 biological processes are involved in drought stress defense in drought-tolerant material and drought-sensitive material materials, respectively (Fig. 2A, B). Most DEGs related to biological processes were enriched in terms of protein phosphorylation, response to oxygen-containing compounds, and response to endogenous stimulus in the tolerant material to drought. The single-organism metabolic process, the biosynthetic process of organonitrogen compounds, and the metabolic process of small molecules were the most abundant category in the sensitive material to drought (Fig. 2A, B). For the cellular components, terms related to photosynthesis such as thylakoid, thylakoid part, chloroplast thylakoid, plastid thylakoid, photosynthetic membrane, thylakoid membrane, chloroplast thylakoid membrane, plastid thylakoid membrane, photosystem, and chloroplast were involved in response to drought stress in 11, while only two terms (photosystem I and ribosome) were significantly regulated by drought stress in sensitive material (Fig. 2A, B). In terms of molecular function, 481 proteins kinase activity-related genes, 205 transcription factor activity-related genes, and 205 sequence-specific DNA binding, nucleic acid binding, and transcription factor activity-related genes were significantly regulated by drought in drought-tolerant material, while most genes significantly regulated by drought in sensitive material were related to oxidoreductase activity (Fig. 2A, B).

### Key pathway and metabolism process involved in response to drought stress

**Fig. 3:**
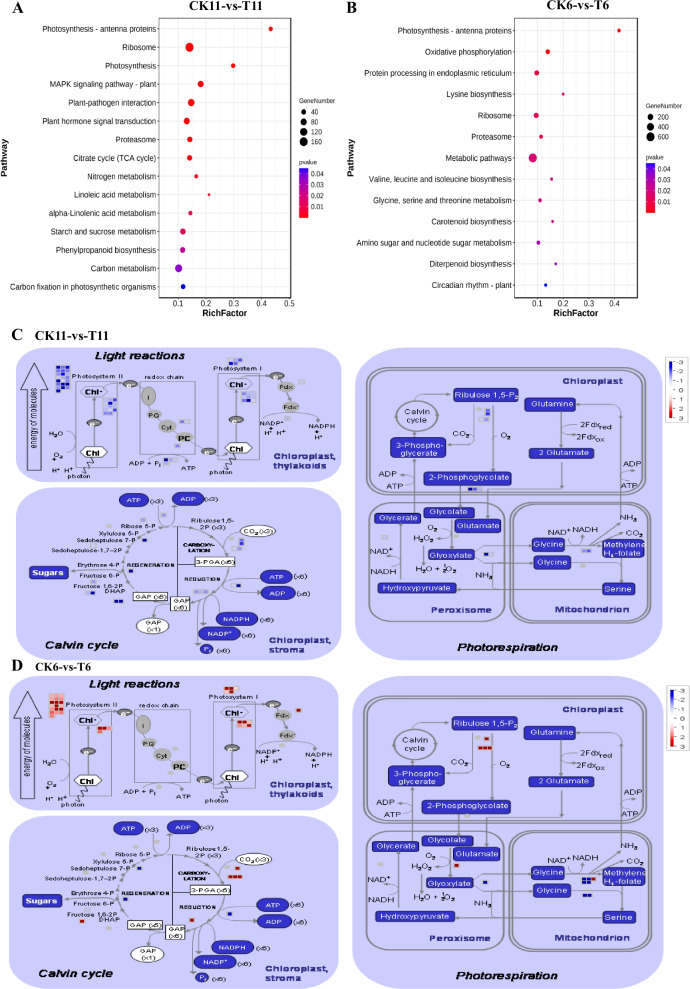
Significantly enriched KEGG pathways and DEGs involved in photosynthesis of *Artemisia wellbyi* under drought stress. **A** Significantly enriched KEGG pathways in drought-tolerant material which were annotated in www.kegg.jp/kegg/kegg1.html. **B** Significantly enriched KEGG pathways in drought-sensitive material. **C** DEGs involved in photosynthesis in drought-tolerant material. **D** DEGs involved in photosynthesis in drought-sensitive material. The size of node indicates the number of DEGs, and the red color represents a lower *p*-value while the blue color represents a higher *p*-value in Fig. 3A–B. The red color represents up-regulated genes while the blue color represents down-regulated genes in Fig. 3C–D. CK11, well-watered drought-tolerant material; T11, drought-tolerant material exposed to drought stress; CK6, well-watered drought-sensitive material; T6, drought-sensitive material exposed to drought stress

KEGG pathway analysis was performed to identify the metabolic pathways that are altered in response to drought stress in *A. wellbyi*. The pathways with *p*-value < 0.05 were considered highly related to defense against drought stress. We observed that signal transduction pathways such as plant hormone signal transduction pathways, the MAPK signaling pathway, and plant–pathogen interaction pathways were significantly enriched in drought-tolerant material (Fig. 3A). In addition, photosynthesis and related pathways such as carbon metabolism, carbon fixation (in photosynthetic organisms), and starch and sucrose metabolism were also involved in defense against drought stress in tolerant material (Fig. 3A). Furthermore, secondary metabolism pathways such as linoleic acid metabolism pathways and phenylpropanoid biosynthesis pathways were also considerably activated after drought stress in tolerant material (Fig. 3A). However, no signal transduction pathway was enriched in sensitive material after drought stress (Fig. 3B). Thirteen significantly enriched KEGG pathways were largely related to primary and secondary metabolism, such as the oxidative phosphorylation pathway, amino acid metabolism pathways, and diterpenoid biosynthesis (Fig. 3B). Photosynthesis-related pathways such as the photosynthesis–antenna proteins pathway and carotenoid biosynthesis pathway were also significantly regulated by drought stress in drought-sensitive material (Fig. 3B).

Given the important role of photosynthesis in responding to drought stress (Figs. 2 and 3), genes involved in the light reaction, Calvin cycle, and photorespiration were further investigated. The result showed that all DEGs related to the light reaction, Calvin cycle, and photorespiration were downregulated in drought-tolerant materials, whereas most of them were upregulated in drought-sensitive materials (Fig. 3C–D). We also found that the P_N_, T_r_, C_i_, and g_s_ of drought-tolerant and -sensitive *A. wellbyi* were reduced by drought, and the P_N_, T_r_, C_i_, and g_s_ of drought-tolerant *A. wellbyi* were significantly lower than drought-sensitive *A. wellbyi* after a 3 d of drought stress (Fig. S1). However, the P_N_, T_r_, C_i_, and g_s_ of drought-tolerant *A. wellbyi* were higher that drought-sensitive *A. wellbyi* after a 30 d of drought stress (Fig. S1). These results showed that the inhibition of photosynthesis by drought stress were due to stomatal closure in drought-tolerant *A. wellbyi*.

### Comparison of transcriptional regulation in response to drought stress between the resistant and sensitive material

**Fig. 4:**
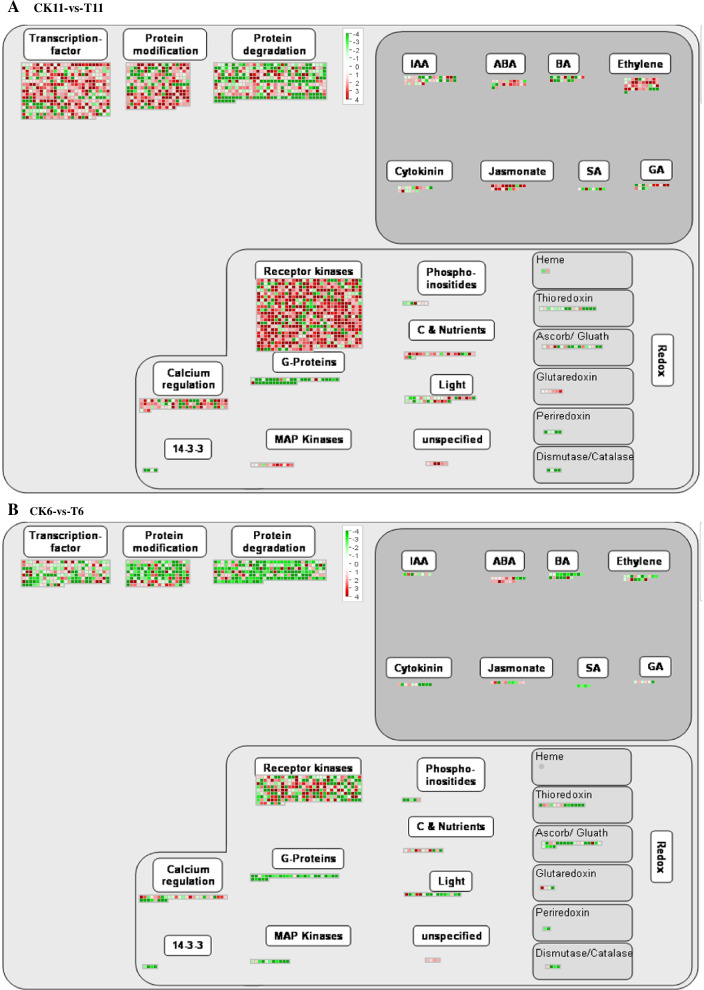
Identification and characterization of the transcriptional regulation responding under drought stress in leaves of *Artemisia wellbyi* between the drought-tolerant material “11” and drought-sensitive material “6”. **A** Transcriptional regulation responding in leaves of drought-tolerant material. **B** Transcriptional regulation responding in leaves of drought-sensitive material. The red color represents up-regulated genes while the green color represents down-regulated genes. CK11, well-watered drought-tolerant material; T11, drought-tolerant material exposed to drought stress; CK6, well-watered drought-sensitive material; T6, drought-sensitive material exposed to drought stress

DEGs induced by drought stress in the leaves of both materials were annotated using MapMan software. The results showed that more transcriptional regulators in the drought-tolerant material “11” were upregulated while downregulated in the drought-sensitive material “6” under drought stress (Fig. 4 A, B). There were 421 and 187 differential expression TFs (DETs) involved in response to drought stress in drought-resistant material “11” and drought-sensitive material “6”, respectively (Fig. 4 A, B). A total of 182 upregulated and 66 down-regulated DEGs were involved in protein modification in drought-tolerant material “11,” whereas only 47 upregulated and 95 down-regulated DEGs were related to protein modification in sensitive material “6” (Fig. 4 A, B). Furthermore, more DEGs were involved in protein degradation in drought-tolerant material “11” than in sensitive material “6” (Fig. 4 A, B). A total of 173 and 89 hormone-related DEGs were involved in response to drought stress in drought resistance and sensitive materials, respectively (Fig. 4 A, B). A total of 41 genes related to ethylene (ET) and 36 auxins (IAA) were significantly enriched and regulated in response to drought stress in drought-tolerant material “11” under drought stress (Fig. 4 A). More receptor kinase genes, calcium regulation genes, and G-protein genes were significantly regulated in the drought-tolerant material (Fig. 4 A, B). Furthermore, MAP kinase genes in the tolerant material were upregulated but down-regulated in sensitive material (Fig. 4 A, B).

### Drought-induced DETs in resistant material

**Fig. 5:**
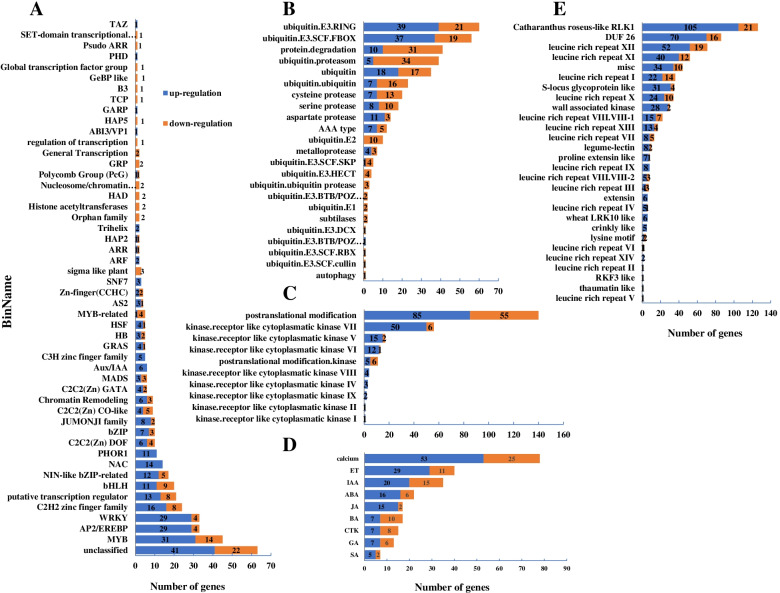
Drought stress-induced differential expression transcriptions factors (DETs, **A**), protein degradation genes (**B**), protein modification genes (**C**), plant hormone and calcium regulation genes (**D**), receptor kinases genes (**E**) in drought tolerant material “11”

To further identify the TFs significantly regulated by drought stress in tolerant material “11”, different DETs were classified into 51 families (Fig. 5A). The results showed that the members of the MYB domain transcription factor family were the most enriched, followed by AP2/EREBP and the WRKY domain transcription factor family after drought stress, including 32 members of the MYB- and MYB-related transcription factor family that were up- or down-regulated, 29 members of AP2/EREBP that were upregulated and four down-regulated, and 29 members of WRKY that were upregulated and four down-regulated (Fig. 5A, Table S5). In addition, 16 up- and eight down-regulated members of the C2H2 zinc finger family, 11 up- and nine down-regulated bHLHs, 12 up- and five down-regulated members of the NIN-like bZIP family, 14 upregulated NACs (NAC domain transcription factor family members) were also involved in response to drought stress in tolerant material “11” (Fig. 5A, Table S5). It should also be noted that the C2C2 (Zn) Dofs, the C2C2 (Zn) CO-like, the C2C2 (Zn) GATAs, and the members of the C3H zinc finger family were regulated by drought stress (Fig. 5A, Table S5). Furthermore, the Aux/IAA regulator and auxin response factor (ARF) were upregulated by drought stress in tolerant material “11” (Fig. 5A, Table S5). More than 10-fold change on expression levels of NAC domain-containing protein 68 (unigene0122553), MYB73 (unigene0054293), WRKY (unigene0001805), and MYB3R-3 (unigene0016830) were induced by drought in drought-tolerant *A. wellbyi*. Overall, these results indicate that a number of TFs in tolerant plants are collectively involved in response to drought stress.

### DEGs involved in protein modification and degradation in tolerant material “11”

Drought-induced protein degradation in the tolerant material was mostly related to the E3 ligase, including the RING finger, the subunit of the SCF ubiquitin ligase complex (F-box, SKP, RBX, and cullin protein), HECT, BTB/POZ, and DCX ubiquitin E3 ligase protein (Fig. 5B, Table S6). 2 F-box ubiquitin E3 ligases, 1RING ubiquitin E3 ligases, and 1 SKD1 (Suppressor of K^+^ Transport Growth Defect1) were up-regulated more than 10-fold change in drought-tolerant *A. wellbyi*. In addition, ubiquitin polyprotein (unigene0092912), Cullin homolog 3 (CUL3; unigene0085305), SUMO liagse (unigene0131876), and Ribosomal protein S27a (unigene0082080) were down-regulated more than 12-fold change. Our results suggest that ubiquitin-mediated proteolysis plays a key role in increased drought tolerance. Differentially expressed protein modification gene analyses indicated that 248 DEGs, including MAPKs, CIPKs (CBL-interacting protein kinases), and PP2Cs (protein phosphatase 2C family proteins) were involved in defense against drought stress in the tolerant material (Fig. 5C, Table S7). Furthermore, receptors such as the subfamily proteins of cytoplasmic kinase I, II, IV, V, VI, VII, VIII, and IX were also significantly regulated by drought (Fig. 5C, Table S7). We also observed that the Ca^2+^-dependent signal transduction pathway genes SOS3 (salt overly sensitive) interacting protein 4 (SIP4), and CDPK were significantly regulated by drought (Table S7). 12 genes belong to receptor like cytoplasmatic kinase V, VI, VII, IX and 3 genes belong to protein kinase superfamily protein were up-regulated more than 10-fold by drought compared with the control in drought-tolerant *A. wellbyi* (Table S7).

### DEGs associated with plant hormone, calcium signaling, and receptor kinase in tolerant material

Under drought conditions, 78 calcium signaling-associated genes were significantly regulated (Fig. 5D). Among these, calmodulin-binding family proteins (CBPs), CDPKs, calcium-binding EF hand family proteins, calcium-transporting ATPases, and calmodulin-like proteins involved in calcium signal transduction (Table S8). There are 40 DEGs associated with ET signaling involved in response to drought stress in the tolerant material (Fig. 5D), 14 of them including proteins of the Fe^2 +^-dependent oxygenase superfamily, 1-aminocyclopropane-1-carboxylate synthases (ACCS), and ACC oxidase, involved in ET synthesis/degradation, 21 of them including ethylene-responsive TFs (EREBP), and ERFs (ERF/AP2B3, ERF1, ERF4, ERF5, ERF9, ERF12) involved in transduction of ET signal, six of them including basic DNA-loop-helix (bHLH) DNA-binding superfamily proteins, DEA (D / H) box RNA helicase family proteins, and multiprotein bridging factor 1B (MBF1B) involved in response to ET signal transduction (Table S8). We also found that 35 DEGs related to IAA were involved in response to drought stress in the tolerant material (Fig. 5D). IAA β-glucosyltransferase and IAA β-D-glucosyltransferase were significantly regulated by drought and involved in IAA metabolism (Table S8). The CYP711A1, transport inhibitor response 1 (TIR1) cytochrome 450 family protein, and the binding of HSP40/DaJ peptide were negatively regulated and played a role in the transduction of IAA signals (Table S8). Furthermore, 28 DEGs, including auxin-responsive family proteins, O-fucosyltransferase family proteins, IAA13, IAA-amido synthetase GH3.1, and members of the small auxin-upregulated RNA (SAUR) protein family were involved in the response of the IAA signal (Table S8). Furthermore, the CYP711A1 gene is also involved in the metabolism of ABA, JA, and BR (Table S8). The highly ABA-induced PP2C gene 2 (HAI2) was upregulated in the drought-tolerant material (Table S8). Genes involved in ABA metabolism, such as UDP glycosyltransferase (UGT), 9-*cis*-epoxycarotenoid dioxygenase, and putative hydroxysteroid dehydrogenase (HSD), were also up/down-regulated (Table S8). It should also be noted that most of the genes associated with JA were upregulated in the drought-tolerant material (Fig. 5D, Table S8). More than 10-fold change on expression levels of ERF (ethylene response factor) subfamily B-3 (unigene0101906) and lipoxygenase 1 (LOX1, unigene0168277) were observed in drought-tolerant *A. wellbyi* (Table S8). Most DEGs were upregulated by drought stress for the receptor kinase, and abundant leucine-rich repeats (LRRs) were detected to respond to drought stress (Fig. 5E, Table S9). The RLK1 and DUF26 subfamily proteins were most abundant after drought stress (Fig. 5E, Table S9). Receptor kinases *Catharanthus roseus*-like RLK1 (unigene0073865), leucine rich repeat X (unigene0002407), DUF 26 (unigene0002407), extension (unigene0002407), and proline extensin like (unigene0002407) appeared the highest expression level to response drought stress in drought-tolerant *A. wellbyi* (Table S9).

### Prediction of protein–protein interactions (PPI) to identify the hub genes in response to drought stress of tolerant material “11”

**Fig. 6:**
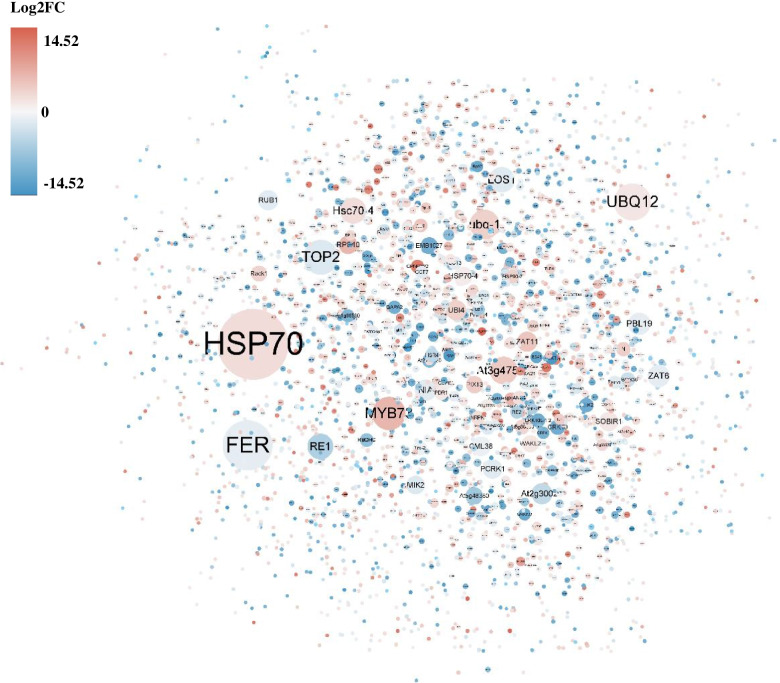
Prediction of interaction relationship between DEGs. Each node represents a DEG, and the bigger size of the node represent more connections. The red color represents an upregulated gene, and the blue color represents a downregulated gene. The deeper the color the larger degree of up/downregulation

Potential interactions between DEGs were further investigated using the String database. The results showed that HSP70 enriched most of the connections followed by receptor-like protein kinase FERONIA (FER), polyubiquitin (UBQ12), topoisomerase 2 (TOP2), MYB73, polyubiquitin1 (ubq-1), At3g47570 (LRR protein kinase family protein), Hsc70-4 (HSP70-like protein), ribonuclease H-like domain-containing protein (RE1), and LOS1 (elongation factor 2-like protein) (Fig. 6). These hub genes play a pivotal role in response to drought stress and downstream gene regulation.

## Discussion

Our study selected a drought-resistant and drought-sensitive *A. wellbyi* from 13 wild materials through the comprehensive assessment of growth characters under drought stress. We found that drought-tolerant material maintains good growth and physiological characteristics under drought stress (Fig. 1C–F). The transcriptome analysis showed that more genes were involved in response to drought stress in the drought-tolerant material (Fig. 1G). DEGs involved in protein phosphorylation, response to oxygen-containing compounds, response to endogenous stimulus, photosynthesis, protein kinase activity, and transcription factor activity were detected in drought-tolerant material under drought stress. In addition, regulation factors such as TFs, post-translational modification genes, plant hormone signaling, calcium signaling, and receptor kinases could play important roles in signal transduction and the drought stress response in tolerant material. The signal transduction genes and downstream genes synergistically compose the drought stress interaction network. The hub genes play a key role in response to drought signal transduction and downstream gene regulation. Therefore, a comprehensive transcriptional regulation and PPI network were investigated in the present study.

### Drought stress inhibits photosynthesis in *A. wellbyi*; genes associated with sucrose and starch degradation in drought-tolerant materials were upregulated by drought

Generally, the lack of water availability and stomatal closure are the main reasons that photosynthesis is inhibited under drought stress [33]. The stomata are closed under drought stress to reduce transpiration-induced water loss [34]. However, regulation behavior inhibits CO_2_ assimilation because of reduced stomatal conductance [35]. In *Saccharum officinarum* L., photosynthesis-related genes in the drought-sensitive genotype were down-regulated but upregulated in the drought-tolerant genotype after drought stress [36]. In the present study, DEGs involved in light response, Calvin cycle, and photorespiration were down-regulated in the tolerant material but upregulated in the sensitive material (Fig. 3C–D, Table S10–11). Transketolase is the major rate-limiting enzyme for the Calvin cycle, and overexpression of transketolase plants displayed slow-growth and chlorotic phenotype [37]. A 11.80 and 3.72-fold down-regulation of transketolase gene in drought-tolerant and -sensitive *A. wellbyi* were observed, respectively (Table S10–11). This result indicating that the negative regulation of transketolase gene might play an important role in responding drought stress. The triosephosphate isomerase (TPI) was down-regulated more than 10-fold suggesting that the CO_2_ assimilation and glycolysis process were limited by drought in drought-tolerant *A. wellbyi* (Table S10–11 [38, 39];). The RWC in the tolerant material was higher than in the sensitive material, indicating that lower inhibition of photosynthesis could lead to greater water loss. The P_N_, T_r_, C_i_, and g_s_ of drought-tolerant *A. wellbyi* were significantly lower than drought-sensitive *A. wellbyi* after a 3 d of drought stress (Fig S1), suggesting that the stomatal closure inhibited the photosynthesis of drought-tolerant *A. wellbyi*. The higher P_N_, T_r_, C_i_, g_s_ and better growth performance of drought-tolerant *A. wellbyi* after 30 d drought stress might be attribute to the faster stomatal closure to reduce the water loss. AbdElgawad [40] reported that starch biosynthesis contributes to defense against drought stress in maize (*Zea mays* L.) by increasing pigment concentrations and ribulose-1,5-bisphosphate carboxylase/oxygenase (RuBisCo) activity. Our results showed that most genes involved in starch and sucrose biosynthesis were down-regulated, indicating that drought-induced photosynthesis inhibition might reduce carbon assimilation in *A. wellbyi* (Table S12–13). The Glucose-1-phosphate adenylyltransferase large subunit 1 was up-regulated for 10.61-fold in drought-tolerant *A. wellbyi* suggesting that drought stress might contribute to the starch biosynthesis (Table S12 [41];). We observed that genes involved in sucrose and starch degradation, such as neutral invertase, cell wall invertases, vacuolar invertases, sucrose synthase activity (SUS3), α-amylase, and β-amylase were upregulated in the tolerant material, whereas most of them were down-regulated in the sensitive material (Table S12–13). The degradation of sucrose and starch in the tolerant materials could be beneficial in alleviating osmotic stress and balancing excess ROS [42].

### Positive transcriptional regulation and phytohormone metabolism play a key role in defense against drought stress

Upon exposure to drought, the ABA-dependent signal transduction pathway, the MAPK signal transduction pathway, the CDPK, and the SOS signal transduction pathway are the first receptors that respond to drought stress in plants [43]. Arabidopsis MAPKKK18-overexpressing seedlings [44], soybean seedlings overexpressing CDPK3 (*Glycine max*) [45], and rice seedlings overexpressing SOS2 [46] had significantly improved tolerance to drought stress. We found that CDPKs (CDPK1, CDPK2, CDPK9), SOS3, and MAPKs (MAPK1, MAPK9, MAPKK2, MAPKKK5, MAPKKK14) were significantly regulated in drought-tolerant material, indicating that the MAPK, CDPK, and SOS signal transduction pathways contribute to improved drought resistance of *A. wellbyi* (Table S8 & S14). 3 calmodulin-binding proteins (unigene0081320, unigene0043259, unigene0041784) were up-regulated by drought more than 5.97-fold change indicating that they might play a pivotal role in enhance drought resistance in drought-tolerant *A. wellbyi* ( Table S8 [47];). Previous studies have shown that the membrane-anchored receptor-like kinase (RLK) family plays a key role in stress signaling transduction through phosphorylation or other mechanisms [48]. We observed that 478 DEGs involved in protein phosphorylation through GO classification were mostly related to the protein kinase superfamily protein (Fig. 2A, Table S15). Mapman analysis showed that most receptor kinases were upregulated in the drought-tolerant material (Fig. 4A); The leucine-rich repeat (LRR) protein kinase family protein and *Catharanthus roseus*-like RLK1 associated protein were mostly enriched after drought stress (Fig. 5E, Table S9). DEGs associated with receptor kinases are characterized mainly by the repeated leucine and cysteine-rich domain (Table S9). Furthermore, drought also induced RLK, HERCULES1 (HERK1), and LRR-RLK. These receptor kinases could directly bind to TF or mediate the biosynthesis of plant hormones to improve drought tolerance [49]. More than 11-fold changes on expression levels of receptor kinases *Catharanthus roseus*-like RLK1 (unigene0073865), leucine rich repeat X (unigene0002407), DUF 26 (unigene0002407), extension (unigene0002407), and proline extensin like (unigene0002407) were induced by drought indicating that the significant positive regulation of these receptor kinases could play a key role in drought stress defense in *A. wellbyi*.

TFs have been widely reported to be the main regulator of gene expression under drought stress [50]. As mentioned in the introduction, numerous TFs such as MYB [51], NAC [50], WRKY [52], bZIP [19], and bHLH [24] are induced by drought. In *Arabidopsis,* the *AtMYB60* gene can specifically regulate stomatal movements under drought stress [53]. Moreover, overexpression of MYB73 TF can enhance plant salt tolerance [54]. We found that 50 MYB TFs were significantly induced by drought in drought-tolerant material (Table S5), and the PPI network showed that MYB73 interact with 1778 downstream DEGs (Fig. 6, Table S16). A 11.82-fold change on expression level of MYB 73 (unigene0054293) was induced by drought in drought-tolerant *A. wellbyi*. This result highlights the critical role of MYBs in responding to drought stress. Previous studies have shown that AP2/EREBP TFs play an important role in improving plant drought resistance. SHINE2 from the AP2/ EREBP family can regulate the wax content and composition of the apple epidermis to improve resistance to drought [55]. EREB160 can enhance plant drought tolerance by regulating genes related to the ABA signaling pathway [56]. Some AP2/EREBP TFs are also controlled by ET and JA under abiotic stress [57]. We observed that a total of 29 genes in the DREB subfamily and genes and ERF subfamily were upregulated by drought stress in tolerant material (Fig. 5, Table S5). The DREB subfamily A-5 (unigene0131917), CBF4 (C-repeat/dehydration-responsive element binding factor4, unigene0021371), and DREB subfamily A-1 (unigene0054014) were up-regulated more than 6-fold change compared with the control in drought-tolerant *A. wellbyi*. We also found that genes involved in ET metabolism such as ACSs and ACC oxidase, and responses such as the bHLH and DEA (D/H) box RNA helicase family protein were induced by drought (Table S8). These results suggest that ET signaling is also involved in the response to drought stress in tolerant material.

A strong relationship between phytohormones and the drought resistance of plants has been reported in previous literature [58]. Salvi [59] showed that the essence of drought stress reactions in plants is driven by phytohormones and their regulated metabolism pathways. There is evidence that ABA plays a crucial role in regulating TFs and downstream stress-responsive genes. A *de novo* biosynthesis of ABA would be induced under drought stress to enhance the drought resistance of the plant [58]. The genes involved in ABA metabolism, such as 9-cis-epoxycarotenoid dioxygenase, signal transduction such as ABA insensitive 1 (ABI1), and responses such as the highly ABA-induced PP2C gene 3 (HAI3), were upregulated in the tolerant material (Table S8). The expression level of 9-cis-epoxycarotenoid dioxygenase (unigene0079175) was increased more than 3-fold suggesting that drought-tolerant *A. wellbyi* could accumulate the ABA to enhance drought-resistance [60]. Furthermore, auxin-related genes also induced by drought develop the lateral root of plants [61]. We observed that the auxin response regulator and ARF, Aux/IAA family genes, and SAUR-like auxin-responsive protein family genes, GH3 and GH3.1, were induced by drought (Table S8). In addition, it was reported that a cytochrome P450 gene CYPM1 negatively regulated by osmotic stress and play a pivotal in auxin transportation [62]. Our result found that the CYP711A1 (unigene0009397) was also down-regulated by drought stress in drought-tolerant *A. wellbyi* more than 11-fold change on expression level. These results suggest that ABA and IAA signaling involved in enhancing drought tolerance in tolerant material.

### Post-translational modification plays a critical role in determining drought tolerance

Post-translational modification is also considered a major pathway involved in the defense against drought stress. Ubiquitylation is a crucial process that is associated with protein-specific degradation [63]. Previous studies have shown that U-box and F-box E3 ubiquitin ligase genes play a central role in regulating stomatal closure [64] and ABA signaling [65, 66]. We found that 18 RING/U-box superfamily genes and 56 F-box family genes were significantly regulated by drought (Table S6). A F-box E3 ubiquitin ligase At1g08710 of Arabidopsis can enhance drought resistance through a negative regulation mechanism [67]. However, our study showed that the F-box E3 ubiquitin ligases (unigene0128583 and unigene0072177) were up-regulated more than 10-fold by drought in drought-tolerant *A. wellbyi*. The ubiquitylation-induced modifications of HSP70 also play a crucial role in improving drought tolerance [63]. We found that HSP70 enriched most downstream genes and ubiquitin ligase proteins, including F-box family proteins and U-box family proteins, involved in regulating HSP70 (Fig. 6, Table S17). More that 14-fold change on expression level of HSP70 were observed in drought-tolerant *A. wellbyi* showed which might play a critical role in responding drought stress (Table S17). Furthermore, SnRK2, MAPKs, CIPKs, ABIs, and LRR receptor kinases were observed and are involved in post-translational modification (Table S7). These results suggest that post-translational modification and the hub gene HSP70 play a key role in responding to drought stress in tolerant material.

## Conclusions

In summary, the positive transcriptional regulation is beneficial to responding drought stress in the *A. wellbyi*. The genes involved in ABA-dependent signal transduction pathway, MAPK signal transduction pathway, and calcium signaling pathway were significantly regulated by drought in the tolerant *A. wellbyi*. MYBs, AP2/ EREBPs, WRKYs and bHLHs were also identified to play a role in enhance drought resistance in the tolerant *A. wellbyi*. Post-transcriptional modification of HSP70 and regulation of MYB 73 might be the hub genes that associated with better growth performance under drought stress. Additionally, negative regulation of photosynthesis in early stage after drought stress in the tolerant *A. wellbyi* might advantage in water retention.

## Materials and methods

### Seed collection and plant culture and treatment

The seeds of *A. wellbyi* were collected in different areas of the Tibetan Plateau (Table 1). We have permission to collect plant material. The voucher specimen, BNU 0040250, was identified by Yi He and its sheet was deposited in the herbaria BNU (http://sweetgum.nybg.org/ih/herbarium.php), and it also could be searched on the Chinese Virtual Herbarium (https://www.cvh.ac.cn/spms/detail.php?id=f96f6165) with using name (*Artemisia wellbyi*) and code (0040250). Based on our previous experiments (unpublished work), a water control experiment was performed to evaluate the drought tolerance of wild materials, and a drought-tolerant material (Rank 11) and drought-sensitive material (Rank 6) were selected from 13 wild *A. wellbyi* materials based on a comprehensive assessment of growth characteristics through the membership function method after drought stress. Two identified *A. wellbyi* materials were used in the present study. The seeds of 11 and 6 were planted in seedling pots and watered every day to provide enough water for germination. At the 3–4 leaf stage, uniform seedlings of 11 and 6 were transplanted into flowerpots (basal diameter: 11.4 cm, top diameter: 21 cm, height: 12 cm), each with 15 plants. The substrates for seedlings cultivation were mixed using nutrition soil, sand, and vermiculite in a 2:1:1 ratio. The establishment period was carried out in a greenhouse with a constant temperature of 22/20 ± 1℃ (day/night) and a 16/8 h day/night photoperiod (600 μmol m^−2^ s^−1^) with relative humidity of 60%. After a 90-day establishment period, the seedlings of 11 and 6 were divided into two groups (CK: well-watered treatment and T: drought stress treatment), respectively. Soil moisture measurement (SU-LPC, Beijing) was used to monitor soil water content. The water content in the 11 and 6 CK groups was maintained at 70 to 80% of the maximum field moisture capacity. The water content was maintained at 10–20% for the T group after continuous evaporation. Before stress application, we well-watered all *A. wellbyi* seedling plants continuously to keep pot soil field capacity with a saturated water-bearing condition., and added the weight difference amount of lost water by utilization and transpiration for the control treatments (CK) to keep 70-80% of the maximum water content in each day, but no watering for the drought stress treatments (T) by a gradual reduction until the soil water was naturally consumed to the soil moisture content of 10–20%. Then the water content of each treatment is maintained within the set range in three days, the leaves of 11 and 6 were collected separately and stored at -80 °C after freezing in liquid nitrogen for RNA-seq.

**Table 1:** The material information of *Artemisia wellbyi*

Code	Drought resistance	Habitat	Longitude and latitude	Altitude (m)
11	Tolerant	Sand in river valley	88°18′E; 29.12′N	3957
6	Sensitive	River shoal	91°4′E; 29°17′N	3569

### Determination of growth and physiological characters of *A. wellbyi* under drought stress

The determination of the characteristics of the phenotypes was carried out after a drought stress period of 30 d. Relative water content (RWC), plant height, malondialdehyde (MDA) content, and relative electrical conductivity (REC) were used to characterize the degree of drought stress. Determination of RWC and MDA were carried out as described by Niu [68]. REC determination was conducted as described by Zhang [69]. To represent the photosynthetic process of *A. wellbyi* from under drought, an automatic photosynthetic measuring apparatus (GFS-3000; Heinz Walz GmbH, Germany) was used to measure the net photosynthetic rate (P_N_), transpiration rate (T_r_), intercellular CO_2_ concentration (C_i_), stomatal conductance (g_s_) of each *A. wellbyi* at 0, 3 and 30d [70], the light intensity was 1400 μmol·m^−2^ ·s^−1^, and the CA (ambient CO_2_) value was 588.3 ± 7.1 μmol·mol^−1^ measured under natural conditions.

### RNA extraction, library construction, and RNA sequencing

A total of 0.2 g of leaf tissue collected from each of the 12 samples was used for RNA extraction [71]. As described by Xie [72], the total RNA was extracted using a Trizol reagent kit (Invitrogen, Carlsbad, CA, USA). The 1% agarose gel method was used to monitor RNA degradation and sample contamination. mRNA was fragmented using a fragmentation buffer after eukaryotic mRNA was enriched using oligo(dT) beads, and rRNA was extracted using a Ribo-ZeroTM magnetic kit (Epicentre, Madison, WI, USA). The first-strand cDNA was synthesized using a M-MuLV reverse transcriptase system. The second-strand cDNA was synthesized using DNA polymerase I, RNase H, dNTP, and a buffer. The QiaQuick PCR extraction kit (Qiagen, Venlo, The Netherlands) was used to purify the cDNA fragments, which were sequenced using the Illumina Novaseq 6000 (Gene Denovo Biotechnology Co., Guangzhou, China). Raw data have been deposited in the National Center for Biotechnology Information (NCBI) under the BioProject accession number PRJNA827352.

### Filtering of clean reads, *de novo* assembly, and annotation

Raw reads from the sequencing machines were further filtered by fastp (version 0.18.0) to obtain clean, high-quality reads [73]. *De novo* read assembly was performed using the Trinity program (version 2.8.4) [74]. The integrity of the assembled transcripts was evaluated with Benchmarking Universal Single-Copy Orthologs software (http://busco.ezlab.org/). Taxonomic and functional annotation were performed as described by Shi [75] and Niu [71].

### Differential expression analysis and functional annotation

The assembled unigenes were quantitated using the RSEM software (version 1.2.19) [76]. DESeq2 software (version 1.20.0) was used to perform differential expression analysis for the CK and T group [77]. The read counts were normalized, and the *p*-value and false discovery rate (FDR) were calculated. Genes with a parameter of FDR < 0.05 and |log_2_ fold change (log_2_FC)|< 1 were considered to be DEGs. Gene ontology (GO) classification and Kyoto Encyclopedia of Genes and Genomes (KEGG) pathway enrichments were performed for the DEGs to describe the properties of genes and their enriched pathways comprehensively [78, 79]. The DEGs were mapped using MapMan software (version 3.5.1R2) to predict and classify transcriptional regulators such as TF, phytohormone-related genes, kinases, protein modification systems, and degradation genes [80].

### Protein–protein interaction

The DEGs of drought-tolerant material (11) were searched in the String database [81] to identify the drought stress-induced gene expression network. A coexpression gene network was exported, and the interactions between proteins were revealed. Furthermore, the network was visualized using Cytoscape software (version 3.8.2), and the core and hub genes were presented [82].

### Confirmation of gene expression levels from RNA-seq

Quantitative real-time polymerase chain reaction (qRT-PCR) analysis was performed to confirm the gene expression levels obtained by RNA-seq. Twenty DEGs were randomly selected, and Primer3 software (version 0.4.0) was used to design the primers of selected genes as described by Niu and Ma [71]. The sequences and corresponding genes ID are provided in Table S1. Based on our previous study, the *awHNR* (heterogeneous nuclear ribonucleoprotein) gene was used as a reference gene to normalize the expression alteration in each sample (unpublished work). Three biological and two technical replicates for each treatment were performed to confirm the expression alteration, and the 2^–∆∆CT^ method was used to calculate the expression levels of selected genes [83]. Total RNA was extracted using the RNAsimple total RNA kit (TIANGEN, Beijing, China). The RNA concentration determination and the RNA quality estimation were performed using a spectrophotometer (NanoPro, Tianjin, China). The A260/A280 ratio (1.8–2.0) and A260/A230 (approximately 2.0) were used to select the highly quality RNA samples. The reverse transcription process was performed using the PrimeScriptTM RT reagent kit with gDNA Eraser (RR047A, TaKaRa, Otsu, Japan) in a 20-μL reaction volume. The qRT-PCR and amplification conditions were described by Niu [84].

### Statistical analysis

One-way analysis of variance was used to compare the growth characteristics of drought-tolerant and drought-sensitive material using IBM SPSS Statistics 26 software [85]. GraphPad Prism 9, version 9.0.0 (https://www.graphpad.com) was used to plot the graphs. The data is presented as average value and standard error.

## Supplementary Information

**Additional file 1: Table S1.** Gene ID and sequences of primers.

**Additional file 2: Table S2.** Statistics of filtered data.

**Additional file 3: Table S3.** Statistics of bases data.

**Additional file 4: Table S4. **Statistics of assembly.

**Additional file 5: Table S5. **Drought-induced DETs in the drought-tolerant *A.wellbyi *under drought stress.

**Additional file 6: Table S6 **DEGs involved in protein degradation in the drought-tolerant *A.wellbyi* under drought stress.

**Additional file 7: Table S7. **DEGs involved in protein modification genes in the drought-tolerant *A.wellbyi* under drought stress.

**Additional file 8: Table S8. **Hormone, Ca2+ signalling genes related DEGs in the drought-tolerant *A.wellbyi *under drought stress.

**Additional file 9: Table S9. **Receptor kinases related DEGs significantly regulated by drought.

**Additional file 10: Table S10. **Photosynthesis-related genes in the drought-tolerant *A.wellbyi* under drought stress. 

**Additional file 11:** **Table S11.** Photosynthesis-related genes in the drought-sensitive *A.wellbyi* under drought stress. 

**Additional file 12: Table S12. **DEGs involved in starch and sucrose metabolism in tolerant material.

**Additional file 13: Table S13. **DEGs involved in starch and sucrose metabolism in sensitive material.

**Additional file 14: Table S14. **Differentially expressed MAPKs in drought-tolerant *A.wellbyi*.

**Additional file 15: Table S15. **Protein phosphorylation genes in the drought-tolerant *A.wellbyi *under drought stress.

**Additional file 16: Table S16. **Description and expression levels of PPI network.

**Additional file 17: Table S17. **Interactions between HSP70 and ubiquitination-related genes in the drought-tolerant *A.wellbyi* under drought stress.

**Additional file 18: Table S18. **SRA accession numbers.

**Additional file 19: Fig. S1. **Effects of drought stress on photosynthesis of *A. wellbyi*.

## Data Availability

Transcriptome analysis of *Artemisia wellbyi* under drought stress data generated in this study are released in the NCBI Data Bank (https://www.ncbi.nlm.nih.gov/bioproject/PRJNA827352), the SRA accession numbers are SRR18778726-SRR18778737 (Table S18).

## Abbreviations

DEG: Downregulated differentially expressed gene
MYB: V-myb avian myeloblastosis viral oncogene homolog
NO: Nitric oxide
ABA: Abscisic acid
ET: Ethylene
TF: Transcription factor
DREB: Dehydration-responsive binding protein
bHLH: Basic helix-loop-helix
*AQP*: Aquaporin
*APX*: Ascorbate peroxidase
*SOD*: Superoxide dismutase
*POD*: Peroxidase
*CAT*: Catalase
*Hsp*: Heat shock protein
PPI: Protein–protein interaction
RWC: Relative water content
MDA: Malondialdehyde
REC: Relative electrical conductivity
DET: Differential expression TF
ARF: Auxin response factor
PP2C: Protein phosphatase 2C family proteins
SOS3: Salt overly sensitive
SIP4: SOS3 interacting protein 4
CBP: Calmodulin-binding family proteins
ACC: 1-Aminocyclopropane-1-carboxylate synthase
MBF1B: Multiprotein bridging factor 1B
TIR1: Transport inhibitor response 1
SAUR: Small auxin-upregulated RNA
HAI2: Highly ABA-induced PP2C gene 2
UGT: UDP glycosyltransferase
HSD: Hydroxysteroid dehydrogenase
LRR: Leucine-rich repeat
SUS3: Sucrose synthase activity
RLK: Receptor-like kinase
GO: Gene ontology
KEGG: Kyoto Encyclopedia of Genes and Genomes
