# Supplementary material for: Key factors for differential drought tolerance in two contrasting wild materials of Artemisia wellbyi identified using comparative transcriptomics
Source: BMC Plant Biol. 2022 Sep 17;22:445. doi: 10.1186/s12870-022-03830-3 (PMC9482295; doi:10.1186/s12870-022-03830-3)
Supplement: Supplementary file 1 — Additional file 1: Table S1. Gene ID and sequences of primers. [file 12870_2022_3830_MOESM1_ESM.docx]

| **Gene ID** | **Forward primer** | **Reverse primer** | **Log2FC in RNA-seq** | **Log2FC in RT-qPCR** |  |
| --- | --- | --- | --- | --- | --- |
| Unigene0101906 | ATTCCACAAGGAATGGTGC | CTATGCCAAAACCGGTGAAG | 12.17 | 15.16 |  |
| Unigene0031821 | TATGAAATGTGTCGCGGTTG | CCACTCCAGAAGACGAAGAA | 2.86 | 3.46 |  |
| Unigene0054293 | AAACACGGCCTGGAAAATTG | TAGCCGATCTACCTGGCATA | 11.82 | 8.68 |  |
| Unigene0015222 | CCTAGCAAGGCTAATGCTGA | GAAACAACGTTATCGGAGCC | 10.30 | 8.33 |  |
| Unigene0055398 | AGTCTACACCTTCCAAATGC | GCTCCAATTTATCTCAAGTCCAA | 10.01 | 11.2 |  |
| Unigene0057564 | CAAATGTCGGGATCATGGGA | TCCGAGAATCTGGTTGTGTT | 6.74 | 8.69 |  |
| Unigene0166735 | TTTCACAAGTGGCAGCTATC | CTATCGGTCCCTTTACCCAT | 1.44 | 2.13 |  |
| Unigene0093490 | TTTGGCTTAAGGGTCCAACT | TCCGGTTCTTAACCATCCAG | 3.54 | 5.21 |  |
| Unigene0111049 | GCTGATGCGCTACAACTATG | ACTATCTTGCGCATCTCACA | 9.38 | 7.66 |  |
| Unigene0067303 | TGTGAGATGCGCAAGATAGT | CATTACCATTGCCGATGAGC | 6.66 | 5.14 |  |
| Unigene0044437 | GGTCTCATCCCCTTCGATTT | ACAAGATTTCCATTGCTGCC | 3.65 | 3.44 |  |
| Unigene0087521 | GTTGCACGGGTTTAATTGGA | GGAACTTTCTCTGGGTCGAT | -2.08 | -1.69 |  |
| Unigene0040556 | TACACACTCAAGGTGTCGAG | ACACCATGTCTTGGCCTTTA | -1.54 | -1.31 |  |
| Unigene0168481 | AGTACGAGAGTGTCCCAAAC | GCAGCATCCTCAATGATACC | 9.61 | 8.21 |  |
| Unigene0022128 | GCTCCATGGGACATACAAGA | AACCGTTATCACCACCTTCA | 6.23 | 7.33 |  |
| Unigene0127806 | TGGAATCTTGGCTGTACGAA | ACGTCTCTTGATGGGTTCAC | -11.33 | -15.22 |  |
| Unigene0114185 | GTGGGTCAACTAGGATACCG | CCATACGCAACTGCTTCATC | -12.06 | -10.69 |  |
| Unigene0143362 | TGGCTGAACCCATCAGATTT | ATGGCATAGTCGGTTTGAGT | -7.21 | -5.21 |  |
| Unigene0038920 | CAGATCCGTTTTGCTGACTG | AGCTTGAACCAAGTACACGA | -7.33 | -6.37 |  |
| Unigene0111793 | GAGAGCAAGAAGACCGGTAA | AAGACGACAGGCTTACTGAG | -5.30 | -4.43 |  |
| Pearson’s correlation coefficient (R^2^) | | | 0.9461 | | |

Table S1 Gene ID and sequences of primers
