# Supplementary material for: Key factors for differential drought tolerance in two contrasting wild materials of Artemisia wellbyi identified using comparative transcriptomics
Source: BMC Plant Biol. 2022 Sep 17;22:445. doi: 10.1186/s12870-022-03830-3 (PMC9482295; doi:10.1186/s12870-022-03830-3)
Supplement: Supplementary file 18 — Additional file 18: Table S18. SRA accession numbers. [file 12870_2022_3830_MOESM18_ESM.docx]

**Table S18 SRA accession numbers**

| **Sample name** | **BioProject** | **SRA** |
| --- | --- | --- |
| CK6-1 | PRJNA827352 | SRR18778737 |
| CK6-2 | PRJNA827352 | SRR18778736 |
| CK6-3 | PRJNA827352 | SRR18778733 |
| T6-1 | PRJNA827352 | SRR18778732 |
| T6-2 | PRJNA827352 | SRR18778731 |
| T6-3 | PRJNA827352 | SRR18778730 |
| CK11-1 | PRJNA827352 | SRR18778729 |
| CK11-2 | PRJNA827352 | SRR18778728 |
| CK11-3 | PRJNA827352 | SRR18778727 |
| T11-1 | PRJNA827352 | SRR18778726 |
| T11-2 | PRJNA827352 | SRR18778735 |
| T11-3 | PRJNA827352 | SRR18778734 |
