# Supplementary material for: Key factors for differential drought tolerance in two contrasting wild materials of Artemisia wellbyi identified using comparative transcriptomics
Source: BMC Plant Biol. 2022 Sep 17;22:445. doi: 10.1186/s12870-022-03830-3 (PMC9482295; doi:10.1186/s12870-022-03830-3)
Supplement: Supplementary file 19 — Additional file 19: Fig. S1. Effects of drought stress on photosynthesis of A. wellbyi. [file 12870_2022_3830_MOESM19_ESM.docx]

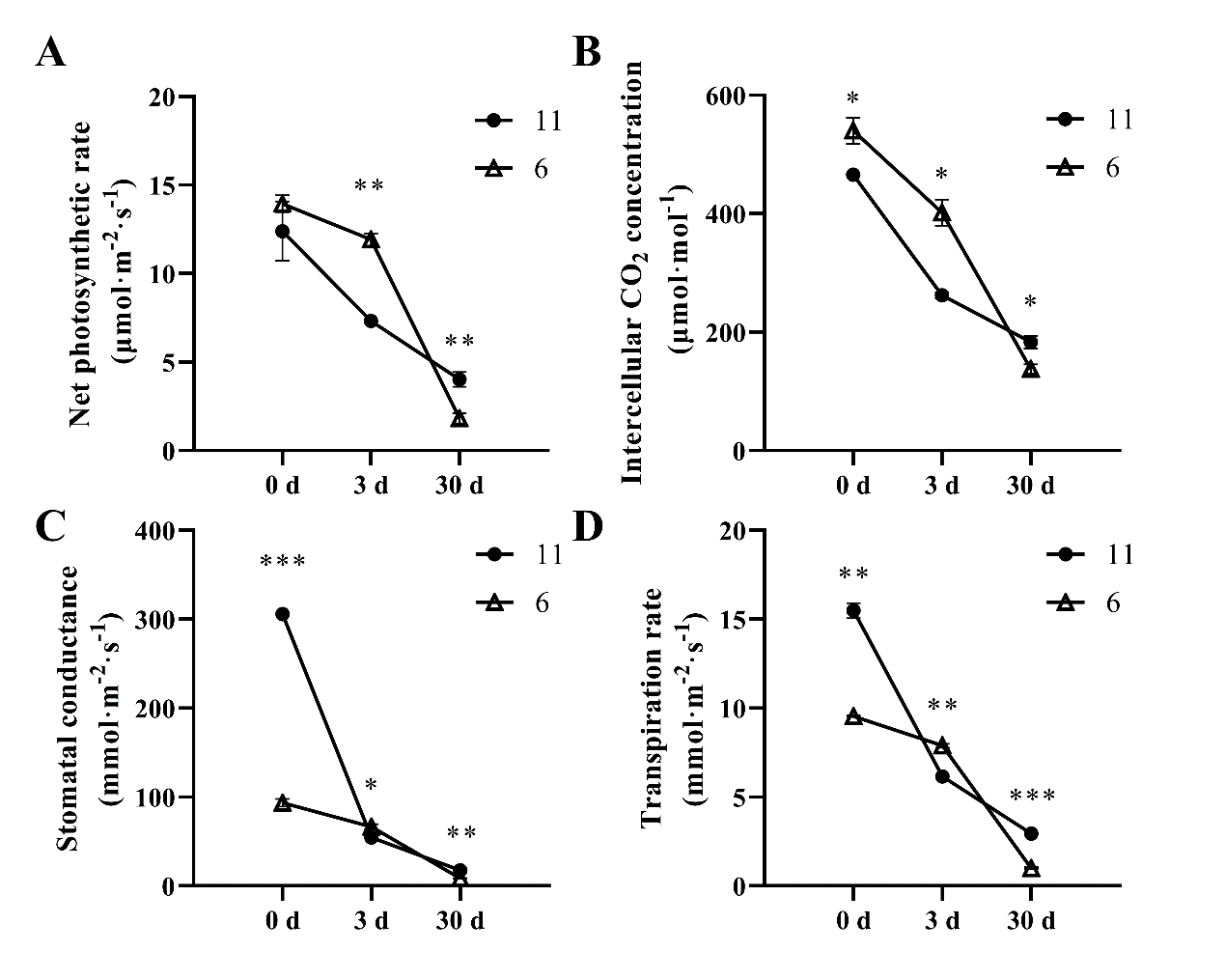


Fig. S1 Effects of drought stress on photosynthesis of *A. wellbyi.* Samples were collected at 0 d, 3 d and 30 d after drought treatment. The significant differences between the drought-tolerant and -sensitive plants were based on Student ’s t-tests at different time points. * *P* < 0.05, ** *P* < 0.01, *** P < 0.001. Data are the means ± SE.
